# Shorter Duration and Lower Quality Sleep Have Widespread Detrimental Effects on Developing Functional Brain Networks in Early Adolescence

**DOI:** 10.1093/texcom/tgab062

**Published:** 2021-10-26

**Authors:** Skylar J Brooks, Eliot S Katz, Catherine Stamoulis

**Keywords:** adolescence, brain, connectome, sleep duration, sleep quality

## Abstract

Sleep is critical for cognitive health, especially during complex developmental periods such as adolescence. However, its effects on maturating brain networks that support cognitive function are only partially understood. We investigated the impact of shorter duration and reduced quality sleep, common stressors during development, on functional network properties in early adolescence—a period of significant neural maturation, using resting-state functional magnetic resonance imaging from 5566 children (median age = 120.0 months; 52.1% females) in the Adolescent Brain Cognitive Development cohort. Decreased sleep duration, increased sleep latency, frequent waking up at night, and sleep-disordered breathing symptoms were associated with lower topological efficiency, flexibility, and robustness of visual, sensorimotor, attention, fronto-parietal control, default-mode and/or limbic networks, and with aberrant changes in the thalamus, basal ganglia, hippocampus, and cerebellum (*P* < 0.05). These widespread effects, many of which were body mass index-independent, suggest that unhealthy sleep in early adolescence may impair neural information processing and integration across incompletely developed networks, potentially leading to deficits in their cognitive correlates, including attention, reward, emotion processing and regulation, memory, and executive control. Shorter sleep duration, frequent snoring, difficulty waking up, and daytime sleepiness had additional detrimental network effects in nonwhite participants, indicating racial disparities in the influence of sleep metrics.

## Introduction

Sleep plays a vital role in maintaining and restoring physiological systems and regulates fundamental processes, such as protein synthesis, hormonal balance, and metabolic waste elimination (Czeisler and Klerman 1999; Mignot, 2008; Imeri and Opp 2009; Robles and Carrol 2011; Xie et al. 2013; Gamble et al. 2014). It is also critical for brain health and has profound effects on cognitive processes, including executive functioning, learning, and memory consolidation (Karni et al. 1994; Stickgold 2005; Diekelmann and Born 2010; Abel et al. 2013). Insufficient and/or poor-quality sleep adversely affects physical, mental, and metabolic health (Van Dongen et al. 2003; Leproult and Van Cauter 2010; Krystal 2012; Schmid et al. 2015) and poses an enormous socioeconomic burden on societies. In the United States of America alone, the associated yearly costs are over ~$400 billion, lost productivity totals more than 1 million working days, and the mortality rate associated with insufficient sleep is at least 10% higher than average (Vgontzas et al. 2010; Hafner et al. 2016). Equally alarming are recent statistics showing that less than half of all children in the United States of America obtain sufficient, high-quality sleep, with about 25% of preschool-age children and over 70% of high-school students sleeping less than the recommended amount for optimal development. Thus, unhealthy sleep is a growing public health problem with serious and often long-term health implications (Jan et al. 2010; Tarokh et al. 2016; Bathory and Tomopoulos 2017).

Across the lifespan, but especially during development, restorative (sufficient and high-quality) sleep plays a fundamental role in cognitive health (Horne 1993; Walker and Stickgold 2004; Ellenbogen 2005; Hobson 2005; Walker 2009; Diekelmann 2014). The development of sensory systems in early life critically depends on sleep (Graven and Browne 2008) as do brain plasticity, learning and memory throughout the first two decades of life (Dang-Vu et al. 2006; Kopacz et al. 2010; Wang et al. 2012). Furthermore, higher level processes, such as decision-making and executive control, continue to develop and are particularly affected by sleep in adolescence (Telzer et al. 2013), as the neural circuitry that supports them undergoes significant maturation during this period.

Prior work has shown that insufficient sleep affects multiple structural and functional brain circuits that support attention, executive function and decision-making, memory, emotional processing and social function, speech and language, sensory processing and motor function (Harrison and Horne 1997; Harrison and Horne 2000; Doran et al. 2001; Drummond and Brown 2001; Jones and Harrison 2001; Durmer and Dinger 2005; Yoo et al. 2007; Alhola and Polo-Kanola 2007; Killgore 2010; Krause et al. 2017; Ben Simon et al. 2020; Váša et al. 2020). Together, these studies highlight profound detrimental effects of insufficient sleep on the brain, including brain regions and networks that undergo significant reorganization during development—particularly in adolescence (Váša et al. 2020), and ultimately cognitive health.

Extensive research has highlighted the importance of sleep specifically in children and young adults and the detrimental effects of sleep loss on cognitive and academic performance, memory, and learning (Dahl 1996; Randazzo et al. 1998; Curcio et al. 2006; Carskardon 2011; Chen et al. 2021). Prior studies have shown that sleep duration is significantly correlated with the developing brain’s structural (white and gray matter) integrity and/or volume (Taki et al. 2012; Telzer et al. 2015). In the short term, structural and functional brain changes associated with insufficient and/or low-quality sleep may lead to extensive negative effects on children’s behavior, daytime sleepiness, fatigue, decreased attention and increased likelihood of risk behaviors. In the long run, repeated sleep loss during development may lead to behavioral, cognitive, and mental health issues in adulthood (Owens, 2014; Tarokh et al. 2016; Medic et al. 2017), possibly as a result of irreversible brain changes.

In addition to quantity, the quality of sleep also plays a significant role in cognitive function across the lifespan. Poor-quality (including fragmented) sleep adversely impacts distributed regions supporting multiple cognitive processes including executive function (Nebes et al. 2009; Harper et al. 2013). Disordered sleep, for example associated with chronic insomnia and sleep-disordered breathing, such as obstructive sleep apnea (OSA) may lead to aberrant structural and functional changes across brain circuits, including orbitofrontal, dorsolateral and ventromedial prefrontal, parietal and occipital cortices, the insula and the amygdala, sensorimotor networks and structures, hippocampal structures and the precuneus (Park et al. 2016; Tahmasian et al. 2016; Chen et al. 2018a, 2018b; Ma et al. 2018). Repeated unhealthy sleep may adversely and in some cases irreversibly affect brain circuits and regions that undergoing neural maturation, leading to long-term cognitive deficits (Beebe 2011; Turnbull et al. 2013). Recent work has shown that sleep-disordered breathing (from snoring to OSA), which affects over 10% of children, may result in reduced gray matter volume and functional network changes in brain regions that support high-level cognitive function and regulate mood (Philby et al. 2017; Chen et al. 2018a, 2018b). Poor-quality sleep may also adversely modulate the developing brain’s functional circuitry, including large-scale networks such as the default mode network (DMN), which play a ubiquitous role in cognitive processing and continue to maturate throughout development (Fair et al. 2008).

Despite extensive prior work on the impact of unhealthy sleep on the brain and cognitive function, there are very few large-sample studies focusing on the impact of sleep on the developing brain and functional circuits that play a fundamental role in cognition. Recent investigations based on children from the Adolescent Brain Cognitive Development (ABCD) study (Casey et al. 2018), which follows almost 12 000 children from preadolescence to young adulthood, have shown increased risk of depression in children who obtain insufficient and/or disturbed sleep (Goldstone et al. 2020) and aberrant brain structure, particularly in regional gray matter in frontal regions, in children who snore (Isaiah et al. 2021). Another study in the same cohort has shown that increased screen time/media use is associated with both decreased sleep duration and reduced quality sleep (Hisler et al. 2020). Finally, poor-quality sleep has also been associated with changes in functional connectivity in distributed brain areas, including the precuneus, anterior cingulate cortex, and lateral orbitofrontal cortex, and overlap with areas that have been linked to depressive symptoms in young adults from the Human Connectome Project (Cheng et al. 2018). These results suggest that insufficient and/or poor-quality sleep may have widespread negative effects on the developing brain and that structural changes are likely to result in detrimental alterations of functional circuits. A few studies in small cohorts have focused on the impact of sleep on the connectivity of specific networks, such as the DMN. Sleep/wake regularity has been associated with increased network connectivity in parts of this network in healthy adolescents and young adults (Lunsford-Avery et al. 2020). In contrast, decreased sleep quality has been related to lower connectivity in the DMN (Tashjian et al. 2018). However, beyond connectivity, the impact of unhealthy sleep on the organization and properties of this and other maturating functional brain circuits remains elusive. Yet, these properties largely determine the brain’s cognitive efficiency, processing speed, ability to integrate information, and resilience across the lifespan. Thus, there is a significant need to systematically investigate the relationship between sleep and the topological organization of functional networks that play a fundamental role in cognition, during complex developmental periods such as adolescence.

In a sample of 5566 typically developing children from the ABCD cohort, this study investigated the impact of sleep duration and quality, assessed via the parent-reported Sleep Disturbance Scale for Children (SDSC), on the resting-state functional connectome in early adolescence, and thus brain networks that play a ubiquitous role in cognitive function across domains. These include the DMN, frontoparietal control, and limbic networks, which continue to maturate throughout adolescence and are thus vulnerable to the effects of risk factors (e.g., unhealthy body mass index [BMI]; Brooks et al. 2021) and stressors such as unhealthy sleep. Given a complex constellation of genetic, environmental, and experiential factors that affect the maturation of functional neural circuitry in the first two decades of life, even the typically developing adolescent connectome is highly variable. Thus, to robustly quantify the effects of unhealthy sleep, a large cohort that captures the population-level heterogeneity of the adolescent brain circuitry, such as the ABCD, is necessary. The SDSC, although subjective, is a widely used tool that facilitates rapid collection of data on sleep quantity and quality in such a large cohort.

Given the ubiquitous role of sleep on brain function, the study hypothesized that reduced sleep duration and/or quality, including increased frequency of disrupted sleep, abnormally modulate local and global topological properties of widespread functional networks, including their efficiency in processing information, robustness/resilience, and the ability to integrate distributed neural computations in response to cognitive demands. To test this hypothesis, the effects of sleep quantity and quality were investigated across multiple spatial scales of the connectome, from individual regions and localized functional circuits to larger networks (Yeo et al. 2011) and the entire brain.

## Materials and Methods

### Participants

All demographic, clinical, survey, and neuroimaging (resting-state (rs) fMRI) data analyzed in the present study were collected at the baseline assessment (study entry) in the ABCD study, a longitudinal investigation of almost 12 000 children. All analyzed data in this study are from Release 2.0.1 and are available through the National Institute of Mental Health Data Archive (NDA 2021). Participant exclusion was based on functional magnetic resonance imaging (fMRI) data quality, imaging using a Philips scanner (preprocessed data prior to Release 3.0 had to be excluded per the ABCD consortium guidelines), and clinical findings in their structural MRI. Additionally, children with a history of bipolar disorder or Attention Deficit Hyperactive Disorder (ADHD) were excluded, given both abnormal functional connectivity and disordered sleep reported, including fragmented sleep, shorter sleep time, and insomnia (Cassoff et al. 2012; Chase and Phillips 2016). Following these exclusions, a total *n* = 5566 children [2669 males (47.95%) and 2896 females (52.08%), 1 missing sex information], median age = 120.0 months (inter-quartile range (IQR) = 13.0 months), range 107–132 months, were included, and all had rs-fMRI of adequate quality for analysis. Almost a third of participants were in early puberty [*n* = 1747 (31.39%)], about a quarter in prepuberty [*n* = 1267 (22.76%)] and a quarter in mid-puberty (*n* = 1427 (25.64%)]. Reflecting the overall race and ethnicity distribution of the ABCD study, the studied cohort was predominantly white [*n* = 3734 (67.09%)] and non-Hispanic [*n* = 4409 (79.21%)]. Combined, participants from all nonwhite race groups (*n* = 1756) accounted for 31.55% of the sample (*n* = 76 (1.35%) were missing data on race). Hispanic participants (*n* = 1097) accounted for 19.71% of the sample (*n* = 60 (1.08%) were missing ethnicity data). Median yearly family income was in the range $75000–$99 000. Over 40.00% of families had a yearly income of ≥$100 000.

### Sleep Duration and Quality Measures

The primary instrument used to assess sleep quality in this study was the parent-reported SDSC (Bruni et al. 1996) with data available for all participants in this study. Parents were asked to report on their child’s sleep in the past 6 months. The SDSC included 26 items to assess multiple aspects of sleep health, including sleep duration, time it takes to fall asleep (sleep latency), and difficulty falling asleep, movement during sleep, excessive sweating, number of times child wakes up from sleep, difficulty falling back to sleep after awakening, difficulty breathing and snoring, teeth grinding, talking and nightmares during sleep, difficulty waking up, and daytime sleepiness. Sleep duration was categorized in the SDSC as: 1 = 9–11 h; 2 = 8–9 h; 3 = 7–8 h; 4 = 5–7 h; 5 = <5 h. Similarly, sleep latency was categorized as: 1 = <15 min; 2 = 15–30 min; 3 = 30–45 min; 4 = 45–60 min; 5 = >60 min. Thus, continuous measurements of sleep duration and latency were not available. All other questions were coded in the SDSC used by the ABCD as: 1 = never; 2 = occasionally (≤1–2 times/month); 3 = sometimes (1–2 times/week); 4 = often (3–5 times/week); 5 = always (daily). Extensive statistical analyses examined the relationships between every item of the SDSC and network measures.

### Additional Variables

I) Caffeine consumption can have a significant impact on sleep quantity and quality. Two measures of caffeine consumption were included in the analyses, a) caffeine use in the past 24 h, a binary variable with 1 = had a caffeinated drink, and b) caffeine use in the past 6 months, measured as the number of drinks per week. This information was extracted from the ABCD Youth Participant Last Use Survey Day 1 2 3 4 (PLUS). Two-hundred forty-nine participants (4.5%) were missing information on weekly caffeine use in the past 6 months. Although surveys completed by youth may be less reliable than those completed by the caregivers, the corresponding parent survey did not include information on caffeine before the fMRI scan (it only included postscan data). Although the SDSC reported sleep habits in the last 6 months and thus only caffeine use in the past 6 months was potentially relevant, information on caffeine consumption in the past 24 h was also included, to assess its impact on the neuroimaging data. A total of 846 children (15.2%) reported consuming caffeine within 24 h prior to their fMRI scan. For 129 participants (2.32%), this information either was missing or was available at a date unrelated to the scan. Over a quarter of all participants, *n* = 1571 (28.22%), had not had any caffeine within the past 6 months. Median caffeine consumption was 0.29 drinks/week (IQR = 1.50).
II) History of anxiety and depression: The Parent Diagnostic Interview for DSM-5 Full (KSADS-5) and Child Behavior Checklist (CBCL) were queried to identify participants with a history of anxiety and/or depression. Although there is substantial debate on whether these mental health issues are contributors or consequences of unhealthy sleep (Alfano et al. 2007; Chorney et al. 2008; Alfano et al. 2009; McMakin and Alfano 2016), there may exist a bidirectional association between sleep and anxiety and/or depression. Thus, the relationship between connectome properties and sleep parameters was adjusted for both. One hundred thirty-five participants (2.43%) had a past history of generalized anxiety disorder, *n* = 406 (7.29%) reported separation anxiety in the past, *n* = 447 (8.03%) reported a past history of clinically significant distress due to social anxiety and *n* = 198 (3.56%) had been diagnosed with a social anxiety disorder in the past. Less than 1% of participants reported any other type of anxiety disorder, and *n* = 89 (1.6%) were missing data for all depression and anxiety categories. A binary variable was then constructed from these data, with 1 = reported anxiety (any type), and 0 = none. A total of 1053 participants (18.92%) reported any type of anxiety. In addition, *n* = 331 (5.95%) reported depressed mood in the past and less than 3% reported any other type of depression. A binary variable was constructed for depression as well, with 1 = any type of depression/depressive symptoms. A total of *n* = 459 (8.25%) children reported any type of depression.
III) Asthma: A number of studies have reported a significant negative association between asthma and sleep quality (Chugh et al. 2006; Van Maanen, 2013) and increased frequency of sleep disordered breathing (Ross et al. 2011). This information was extracted from each participant from the Parent Medical History Questionnaire (MHX). A total of *n* = 893 participants (16.04%) had asthma. Statistical models were adjusted for asthma as a binary (yes/no) variable.
IV) Each participant’s BMI (kg/m^2^) was calculated from height and weight [1 (0.02%) was missing this information] by multiplying weight (measured in pounds) by 703 and dividing by height (measured in inches) squared. In analyses involving subcohorts, age- and sex-specific BMI charts (CDC 2021) were used to estimate a cutoff for obesity, based on which participants were further stratified as obese and nonobese.
V) Screen time included time spent watching television, playing video games and time spent on the mobile phone or computer for any reason (texting, searching the internet, etc.) but excluded time spent on school-related work [four participants (0.08%) were missing this information]. A combined (week plus weekend) average screen time in minutes was estimated for each participant based on the Parent Screen Time Survey (STQ).

### Resting-State fMRI Data Preprocessing

Analyzed neuroimaging data had been only minimally preprocessed by the Data Analysis, Informatics & Resource Center (DAIRC) of the ABCD study (Hagler et al. 2019). The Next-Generation-Neural-Data-Analysis (NGNDA 2021) platform, recently developed for analyses of high-dimensional brain data, was used to further processed the fMRI data, reduce its dimensionality, and denoise it prior to the estimation of the connectome and its properties. FMRI processing using the NGNDA, which uses a combination of widely available and custom-developed computational tools and algorithms, is described in detail in Brooks et al. 2021 and Supplementary Materials. Although each participant had data from up to four (5-min long) (rs)-fMRI runs, only runs with a relatively small number of frames censored for motion (≤10% of total frames) were analyzed. Each participant’s fMRI data were first processed for initial frame removal, coregistration to MRI, slice-time correction, and normalization to MNI152 space, followed by time series denoising and artifact suppression. Then, the spatial dimension of the original fMRI time series matrix was reduced through parcellation to 1088 cortical and subcortical regions and the cerebellum, using a combination of three atlases as described in Brooks et al. (2021). Individual voxel time series within each region were averaged. Since data were acquired with different scanners, all time series amplitudes were normalized by the median absolute amplitude of all regions, in order to be comparable between brains.

### Connectivity and Network Properties Estimation

Only one rs-fMRI run per participant was selected for further analysis, based on the associated connectivity matrix and percent of motion-censored frames. Functional connectivity at rest is typically low across most networks, with the exception of primarily the DMN. Therefore, the run with the lowest median connectivity was selected, which in most brains was also the run with the lowest number of motion-censored frames. Although the NGNDA pipeline provides multiple choices for connectivity estimation, in this study reported results are based on the peak pairwise cross-correlation between fMRI time series, resulting in a 1088 × 1088 symmetric matrix. For comparison, mutual information was also estimated. Resulting connectivity matrices from the two methods had overall statistically identical spatial patterns. These matrices were subsequently thresholded (as described in Brooks et al. [2021] and Supplemental Materials) to obtain weighted and binary adjacency matrices, which were used to estimate network properties.

Network properties were the analyzed outcomes, and were estimated for the entire brain (based on its global topology), the resting-state networks identified in Yeo et al. (2011) based on a high-resolution parcellation of the cortex, with additional separate subcortical and cerebellar networks, and individual brain regions (based on local network node topologies). These properties included network-wide median connectivity, community structure (measured by modularity, local, and global clustering coefficients), global efficiency, small-worldness, network robustness (measured by natural connectivity; Wu et al. 2011), topological network stability (using the largest eigenvalue of the adjacency matrix as a proxy for it), as well as node eigenvalue centrality (measuring the topological importance of a brain region) and degree (the number of a node’s connections). A combination of algorithms in the Brain Connectivity Toolbox (Rubinov and Sporns 2010) and additional ones in NGNDA platform were used in these estimations. Although modularity was primarily estimated using the Newman method (Newman 2006), a second set was obtained using the Louvain method (Blondel et al. 2008). For individual networks, median connectivity was estimated within each network as well as between nodes belonging to the network and those outside the network.

### Statistical Analysis

Statistical analyses of connectome properties were conducted at three spatial levels as well as three cohort levels. First, data from the entire cohort were analyzed (level 1). However, because of significant confounding effects of race and BMI, subcohorts (level 2) corresponding to white and nonwhite or nonobese (*n* = 4802) participants were also analyzed. The nonobese subcohort was separately examined in order to decouple the potential impact of unhealthy weight on sleep duration and quality. Finally, subcohorts of white and nonobese (*n* = 3396) and nonwhite and nonobese (*n* = 1406) were also analyzed, to decouple confounding effects of race and BMI on sleep measures (level 3).

Across levels, ordinary linear regression models were developed to investigate the association between network/node properties (the outcomes) and each sleep variable from the SDSC (the independent variables), while adjusting for potential covariates/confounders. All models were adjusted for age, sex, race [when the entire cohort was analyzed; modeled as a dichotomous variable (1 = white, 0 = nonwhite)], ethnicity (1 = Hispanic, 0 = non-Hispanic), family income, with additional parameters (in separate models) for caffeine consumption in the last 6 months and the past 24 h, anxiety, depression, asthma. To account for sampling differences between sites, propensity weights were included as recommended by the ABCD consortium. Given the distribution of racial identities in this sample, only a dichotomous representation that included all nonwhite participants in the same stratum led to model convergence. All models also included an adjustment for percent of motion-censored frames in the analyzed fMRI run. Although, based on the inclusion criteria for fMRI quality, the selected run had a small number of censored frames (median = 1.90%), in prior work this parameter was found to be significant in statistical models, although its inclusion did not alter the overall results (Brooks et al. 2021). Finally, because pubertal stage information was missing for a more substantial number of participants [*n* = 1020 (18.33%)], sets of models were tested with (using imputation for missing data) and without the inclusion of pubertal stage, using the ABCD ordinal scale: 1 = prepuberty, 2 = early puberty, 3 = midpuberty, 4 = late puberty, and 5 = postpuberty. Across analyses, the significance level was set at *α* = 0.05. Due to the large number of developed models, all *P*-values reported in the text and tables have been adjusted for false discovery rate (FDR) (Benjamini and Hochberg 1995). The model set used to control for FDR varied between analyses. For models testing associations between demographics and the 26 items of the SDSC, the adjustments were made for the 26 *P*-values per independent variable. For models testing associations between individual sleep variables and network properties for the entire brain or subnetworks, the adjustments were made separately for each variable and network for the *P*-values corresponding to all estimated properties. For models testing associations between sleep variables and individual node properties, adjustments were made separately for each variable, adjust *P*-values across nodes within a particular network. The software MATLAB (R2019a, Mathworks, Inc.) was used for all analyses. Figures were generated with the software MRICroGL (NITRC.org).

Separate multivariate models were tested for individual sleep variables. Collinearity between independent variables was assessed before augmenting models with additional parameters. Although multiple strategies were explored for developing models and parameters were shuffled to minimize the final model’s dependence on order of parameter inclusion, the final models reported here included a fixed number of parameters across outcomes so that models can be comparable. The results for the primary sleep variable were statistically identical across modeling approaches. Model fit was assessed using the adjusted *R*^2^ and Akaike Information Criterion (AIC) estimators.

## Results

### Sleep as a Function of Demographics, BMI, and Screen Time

Sleep duration and quality measures were first assessed as a function of non-neuroimaging parameters.

I) Sleep duration: Older children and/or at a more advanced pubertal stage slept less (*P* < 0.01, Wald statistic > 12.0) and had increased sleep latency asleep (*P* = 0.03, Wald statistic = 4.80 for age; the pubertal stage association was non-significant for latency). Girls slept less than boys (median = 8–9 h vs. 9–11 h; *P* = 0.03, Wald statistic = 4.57) and had longer sleep latency (*P* < 0.01, Wald statistic = 10.42). In addition, nonwhite youth slept less than white (median = 8–9 h vs. 9–11 h; *P* < 0.01, Wald statistic = 105.93) and, similarly, Hispanic youth slept less than nonHispanic (median 8–9 h vs. 9–11 h; *P* < 0.01, Wald statistic = 10.14). There was no significant association between race and sleep latency (*P* = 0.21) but Hispanic youth fell asleep faster than nonHispanic (*P* = 0.02, Wald statistic = 5.46). Finally, higher family income was significantly associated with longer sleep (*P* < 0.01, Wald statistic = 139.97) and shorter sleep latency (*P* = 0.02, Wald statistic = 5.27), while higher BMI and longer screen time were significantly associated with shorter sleep (*P* < 0.01, Wald statistic = 29.47 for BMI and *P* < 0.01, Wald statistic = 83.60 for screen time). In addition, the odds of shorter sleep was 1.16 times higher for obese participants (*n* = 764) compared with nonobese (*P* < 0.01). Longer screen time was also significantly associated with increased sleep latency (*P* < 0.01, Wald statistic = 14.80). The amount of weekly caffeine consumed was negatively associated with sleep duration (*P* < 0.01) but no other sleep variable. History of depression and/or anxiety and asthma were significantly associated lower sleep duration (*P* < 0.05). Asthma was significantly associated with increased frequency of snoring and difficulty breathing (*P* < 0.01).
II) Sleep quality: There were multiple significant associations between measures of sleep quality and demographic data, BMI, and screen time. The results are summarized in Table 1 for measures where at least 100 participants reported at least 1–2 events per week (corresponding to a subsample that would have sufficient power to detect statistically differences in a model with 7–8 independent variables). Over 30% of participants went to bed reluctantly at least 1–2 times per week [*n* = 1766 (31.73%)], ~20% had difficulty getting to sleep [*n* = 1038 (18.65%)], ~15% frequently twitched legs while asleep [*n* = 805 (14.46%)], and a similar fraction snored [*n* = 815 (14.64%)]. Almost 20% awakened feeling tired [*n* = 978 (17.57%)] and over 10% had difficulty waking up in the morning [*n* = 643 (11.55%)]. Increased screen time was associated with increased frequency of sleep disturbances (*P* ≤ 0.04) and difficulty waking up, as well as daytime sleepiness (*P* < 0.01). Higher BMI was associated with more frequent movement during sleep, excessive sweating, and snoring (*P* ≤ 0.03), as well as difficulty waking up in the morning and daytime sleepiness (*P* ≤ 0.02). The interaction between BMI and screen time was associated with frequency of almost all adverse sleep quality events (*P* < 0.01). Finally, girls were more often anxious/afraid when falling asleep (*P* = 0.02), had more difficulty waking up, were more frequently tired when waking up and more often sleepy during the day than boys (*P* ≤ 0.04). Younger participants were more frequently reluctant to go to bed, were anxious/afraid when falling asleep sweated excessively, twitched/jerked their legs, and snored (*P* ≤ 0.04). Once corrected for false discovery, no sleep quality measure was significantly associated with pubertal stage (even when age and/or BMI were excluded because of collinearity).

**Table 1:** Model statistics for associations between sleep quality measures [all measured on a scale of 1 (never) to 5 (always)], demographic data, BMI and screen time. The direction of association and corresponding *P*-values (all adjusted for false discovery) are reported. For categorical variables, the category initial is also included with the sign of the regression coefficient, with b = boys, g = girls, w = white, h = Hispanic. All questions refer to the child

	*N* (%) ≥ 1–2 times/week	Age	Sex	Race (White/Non-White)	Ethnicity	Family income	BMI	Screen time
Goes to bed reluctantly	1766 (31.73)	(−) 0.03	—	—	(−h) < 0.01	—	—	(+) < 0.01
Has difficulty getting to sleep at night	1038 (18.65)	—	—	(+w) <0.01	(−h) < 0.01	—	—	(+) < 0.01
Anxious/afraid when falling asleep	351 (6.31)	(−) 0.02	(+g) 0.02	(+w) < 0.01	—	—	—	—
Jerks body while falling asleep	292 (5.25)	—	—	—	—	(+) 0.02	(+) 0.03	(+) 0.03
Experiences vivid dream-like scenes while falling asleep	227 (4.08)	—	—	—	(−h) 0.04	—	—	(+) < 0.01
Sweats excessively while falling sleep	196 (3.52)	(−) 0.04	(+b) < 0.01	—	—	—	(+) < 0.01	—
Wakes up more than twice/night	251 (4.51)	—	—	—	(−h) 0.02	—	—	—
After waking up has difficulty to fall asleep again	182 (3.27)	—	—	—	(−h) < 0.01	—	—	(+) 0.04
Frequently twitches/jerks legs while asleep	805 (14.46)	(−) 0.03	—	—	—	(+) 0.01	—	—
Snores	815 (14.64)	(−) 0.03	—	(−w) <0.01	(−h) <0.01	(−) <0.01	(+) <0.01	(+) 0.01
Sweats excessively during night	306 (5.50)	(−) 0.02	(b+) <0.01	—	—	—	(+) <0.01	(+) 0.04
Talks in sleep	498 (8.95)	—	—	—	—	(−) <0.01	—	(+) 0.02
Grinds teeth during sleep	581 (10.44)	—	—	—	—	—	—	—
Difficulty waking up in the morning	643 (11.55)	—	(+g) 0.04	—	—	(−) <0.01	(+) 0.03	(+) <0.01
Awakes feeling tired	978 (17.57)	—	(+g) <0.01	—	(−h) <0.01	—	—	(+) <0.01
Unable to move when waking up	101 (1.81)	—	—	—	—	(−) <0.01	—	—
Daytime sleepiness	326 (5.86)	—	(+g) 0.02	—	(−h) <0.01	(−) <0.01	(+) 0.02	(+) <0.01

### Effects of Sleep Duration and Quality on Whole-Brain Network Properties

I) Sleep duration had significant effects on network efficiency, global clustering, and modular organization. In the entire cohort, shorter sleep duration was associated with lower connectome efficiency and global clustering (*P* ≤ 0.04, Wald statistic > 5.40) but higher modularity and small-worldness (*P* ≤ 0.04, Wald statistic > 5.50), the latter potentially reflecting developmental effects associated with shorter sleep duration. In the nonobese subcohort, shorter sleep duration was associated with lower network efficiency (*P* = 0.04, Wald statistic = 6.70).
II) Sleep quality: Frequent difficulty breathing during the night (but not snoring) was also significantly associated with lower connectome efficiency and global clustering (*P* = 0.05, Wald statistic > 5.80), and snoring was also associated with lower topological robustness and stability (*P* ≤ 0.020, Wald statistic > 6.14) in nonwhite but not in white participants. Finally, frequent teeth grinding was associated with overall higher median connectivity in the entire cohort (*P* = 0.02, Wald statistic = 9.62) and nonobese participants (*P* = 0.05, Wald statistic = 7.90). No other sleep quality measure had a significant effect on whole-brain network properties or any other subcohort. Neither caffeine consumption before scan nor caffeine consumption over the last 6 months had a significant effect on connectome properties or were statistically significant adjustments (*P* > 0.19 and *P* > 0.44, respectively). The effects of history of anxiety and/or depression were also nonsignificant in these models (anxiety *P* > 0.86; depression *P* > 0.90). The adjustment for asthma was significant only in models testing the association between sleep duration and whole-brain modularity (*P* = 0.03) or small worldness (*P* = 0.01) and in the model testing the association between snoring and modularity (*P* = 0.012)

### Effects of Sleep Duration and Quality on Properties of Individual Networks

Brain regions/nodes were grouped into resting-state networks as described by Yeo et al. (2011).

I) Sleep duration: In the entire cohort (Table 2A), shorter sleep duration was associated with decreased efficiency, global clustering, topological robustness, and stability of bilateral visual (central and peripheral), dorsal and ventral attention, fronto-parietal control and DMNs (*P* ≤ 0.02). Median connectivity within left DMN, left central visual network, right dorsal and ventral attention, and right fronto-parietal control networks also decreased as a function of shorter sleep duration (*P* ≤ 0.04). Median connectivity between regions of the dorsal attention network and the rest of the brain was also negatively affected bilaterally (*P* ≤ 0.03). The only positive association was estimated for modularity, possibly a developmental effect given that modularity increases with age and sleep duration decreases (although all models had been adjusted for age). In assessments of the relationship between sleep duration and individual network properties, the adjustment for asthma was significant in models for efficiency, robustness, and topological stability in the left dorsal attention, right ventral attention/salience, and bilateral control and DMNs (*P* < 0.04).

**Table 2A:** Parameters of models assessing the impact of sleep duration on individual network properties in the entire cohort (*n* = 5566). Regression coefficients have been standardized to allow for comparisons between measures. *P*-values have been adjusted for false discovery. Connectivity is reported within each network (in) and between a network’s nodes and the rest of the brain (out)

Network	Parameter	Efficiency	Global Clustering	Median Conn. (in)	Median Conn. (out)	Modularity	Topological Robustness	Topological Stability
Left hemisphere								
Central visual	Beta	−0.043	−0.039	−0.033	—	0.041	−0.04	−0.041
	*P*-value	0.020	0.020	0.036	—	0.021	0.020	0.022
Peripheral visual	Beta	−0.049	−0.047	—	—	0.061	−0.055	−0.052
	*P*-value	0.002	0.002	—	—	<0.001	0.001	0.002
Dorsal attention	Beta	−0.057	−0.053	—	−0.037	0.054	−0.050	−0.049
	*P*-value	0.001	0.001	—	0.020	0.001	0.001	0.002
Ventral attention/salience	Beta	−0.044	−0.041	—	—	0.055	−0.041	−0.041
	*P*-value	0.009	0.009	—	—	0.002	0.009	0.009
Fronto-parietal control	Beta	−0.044	−0.044	—	—	0.042	−0.038	−0.038
	*P*-value	0.012	0.012	—	—	0.012	0.018	0.018
Default mode	Beta	−0.053	−0.056	−0.056	—	0.051	−0.052	−0.052
	*P*-value	0.001	0.001	0.001	—	0.001	0.001	0.001
Right hemisphere								
Central visual	Beta	−0.035	−0.036	—	—	0.053	−0.042	−0.041
	*P*-value	0.030	0.028	—	—	0.005	0.022	0.022
Peripheral visual	Beta	−0.046	−0.046	—	—	0.054	−0.053	−0.051
	*P*-value	0.004	0.004	—	—	0.002	0.002	0.002
Dorsal attention	Beta	−0.063	−0.062	−0.044	−0.035	0.067	−0.061	−0.060
	*P*-value	<0.001	<0.001	0.005	0.026	<0.001	<0.001	<0.001
Ventral attention/salience	Beta	−0.054	−0.050	−0.039	—	0.065	−0.053	−0.052
	*P*-value	0.001	0.001	0.011	—	<0.001	0.001	0.001
Fronto-parietal control	Beta	−0.055	−0.054	−0.044	—	0.046	−0.051	−0.050
	*P*-value	0.001	0.001	0.005	—	0.002	0.001	0.001
Default mode	Beta	−0.040	−0.045	—	—	0.036	−0.037	−0.038
	*P*-value	0.023	0.022	—	—	0.029	0.023	0.023

To decouple some of the effects of unhealthy BMI, these relationships were also examined in the nonobese subcohort (Table 2B), given that obese youth slept 1–2 h less than nonobese. Similar negative associations were estimated in all but the left fronto-parietal network but also additional decreased median in- and out-of-network connectivity in the right peripheral visual network as a function of shorter sleep duration (*P* ≤ 0.05). Given a significant race effect on this sleep metric, its impact on network properties was examined in white versus nonwhite subcohorts (results for nonwhite are summarized in Table 2C). Similar associations in the same networks were identified, with the addition of a significant negative effect of shorter sleep duration on median connectivity within the left fronto-parietal network (*P* = 0.02) but nonsignificant effects in median in-network connectivity within the right ventral attention network and out-of-network connectivity in the right dorsal attention network. Most of these effects were nonsignificant in the white subcohort. Lastly, sleep duration effects were also examined in the nonwhite and nonobese subcohort (*n* = 1406; Table 2D). Overall similar but also additional negative effects of shorter sleep duration were estimated on properties of the left limbic network (*P* < 0.05) and right DMN (*P* = 0.04), but nonsignificant effects on those of bilateral ventral attention networks. In contrast, in white nonobese participants, there were no significant associations between sleep duration and any of these properties. Throughout these models, adjustments for caffeine and history of anxiety and/or depression were nonsignificant and the adjustment for asthma was significant for models testing the association between sleep duration and efficiency, global clustering, robustness, and stability in left dorsal attention, right ventral attention/salience, bilateral control, and DMNs (*P* ≤ 0.04).

II) Sleep quality: increased difficulty getting to sleep was associated with decreased efficiency, global clustering, median within-network connectivity, topological robustness and stability, bilaterally in the somatomotor and right limbic networks (*P* < 0.05). It also had bilateral negative effects on median connectivity between nodes of somatomotor networks and the rest of the brain (*P* < 0.02), and similarly for the right limbic network (*P* = 0.04). In the nonobese subcohort, similar bilateral negative effects were estimated in the somatomotor network (*P* < 0.02). These results are summarized in Table 3(A-B). Frequent difficulty getting to bed had similar effects on the somatomotor network bilaterally (*P* < 0.03) in white participants (*P* < 0.03) as well, but not in nonwhite (*P* > 0.34). The adjustment for caffeine consumption in the past 24 h, history of anxiety and/or depression and asthma were nonsignificant in these models.

**Table 2B:** Parameters of models assessing the impact of sleep duration on properties of individual networks in the nonobese subcohort (*n* = 4802). Regression coefficients have been standardized to allow for comparisons between measures and *P*-values have been adjusted for false discovery

Network	Parameter	Efficiency	Global clustering	Median conn. (in)	Median conn. (out)	Modularity	Topological robustness	Topological stability
Left hemisphere								
Central visual	Beta	−0.048	−0.044	−0.039	—	0.047	−0.046	−0.047
	*P*-value	0.012	0.015	0.022	—	0.012	0.012	0.012
Peripheral visual	Beta	−0.055	−0.054	—	—	0.069	−0.061	−0.056
	*P*-value	0.002	0.002	—	—	<0.001	0.001	0.002
Dorsal attention	Beta	−0.059	−0.057	—	−0.039	0.048	−0.049	−0.048
	*P*-value	0.002	0.002	—	0.026	0.005	0.005	0.005
Ventral attention/salience	Beta	−0.038	−0.036	—	—	0.045	−0.036	−0.036
	*P*-value	0.042	0.042	—	—	0.035	0.042	0.042
Fronto-parietal control	Beta	−0.041	−0.043	—	—	—	—	—
	*P*-value	0.043	0.043	—	—	—	—	—
Default mode	Beta	−0.054	−0.061	−0.055	—	0.047	−0.050	−0.050
	*P*-value	0.003	0.001	0.003	—	0.004	0.003	0.003
Right hemisphere								
Central visual	Beta	−0.045	−0.047	—	—	0.058	−0.050	−0.049
	*P*-value	0.010	0.010	—	—	0.003	0.009	0.009
Peripheral visual	Beta	−0.055	−0.055	−0.035	−0.033	0.063	−0.062	−0.059
	*P*-value	0.001	0.001	0.040	0.050	0.001	0.001	0.001
Dorsal attention	Beta	−0.062	−0.063	−0.043	−0.038	0.063	−0.058	−0.058
	*P*-value	<0.001	<0.001	0.011	0.025	<0.001	<0.001	<0.001
Ventral attention/salience	Beta	−0.049	−0.043	−0.033	—	0.057	−0.046	−0.046
	*P*-value	0.007	0.010	0.047	—	0.003	0.007	0.007
Fronto-parietal control	Beta	−0.052	−0.055	−0.044	—	0.040	−0.047	−0.047
	*P*-value	0.004	0.004	0.010	—	0.013	0.006	0.006
Default mode	Beta	−0.041	−0.046	—	—	0.036	−0.036	−0.037
	*P*-value	0.041	0.034	—	—	0.041	0.041	0.041

**Table 2C:** Parameters of models assessing the impact of sleep duration on properties of individual networks in the nonwhite subcohort (*n* = 1756). Regression coefficients have been standardized to allow for comparisons between measures and *P*-values have been adjusted for false discovery

Network	Parameter	Efficiency	Global clustering	Median conn. (in)	Median conn. (out)	Modularity	Topological robustness	Topological stability
Left hemisphere								
Central visual	Beta	−0.064	−0.060	−0.061	—	0.069	−0.064	−0.065
	*P*-value	0.025	0.025	0.025	—	0.025	0.025	0.025
Peripheral visual	Beta	−0.062	−0.060	—	—	0.081	−0.068	−0.064
	*P*-value	0.035	0.036	—	—	0.017	0.035	0.035
Dorsal attention	Beta	−0.093	−0.086	—	−0.054	0.100	−0.091	−0.089
	*P*-value	0.001	0.001	—	0.050	0.001	0.001	0.001
Ventral attention/salience	Beta	−0.060	—	—	—	0.076	−0.059	−0.059
	*P*-value	0.043	—	—	—	0.023	0.043	0.043
Fronto-parietal control	Beta	−0.077	−0.079	−0.063	—	0.074	−0.079	−0.079
	*P*-value	0.004	0.004	0.019	—	0.004	0.004	0.004
Default mode	Beta	−0.071	−0.068	−0.072	—	0.082	−0.083	−0.083
	*P*-value	0.009	0.010	0.010	—	0.005	0.005	0.005
Right hemisphere								
Central visual	Beta	—	−0.055	—	—	0.086	−0.069	−0.069
	*P*-value	—	0.048	—	—	0.009	0.027	0.027
Dorsal attention	Beta	−0.099	−0.099	−0.069	—	0.109	−0.102	−0.100
	*P*-value	<0.001	<0.001	0.010	—	<0.001	<0.001	<0.001
Ventral attention/salience	Beta	−0.065	−0.057	—	—	0.065	−0.066	−0.065
	*P*-value	0.021	0.038	—	—	0.021	0.021	0.021
Fronto-parietal control	Beta	−0.083	−0.075	−0.062	—	0.078	−0.084	−0.083
	*P*-value	0.003	0.005	0.021	—	0.004	0.003	0.003
Default mode	Beta	−0.056	−0.056	—	—	0.062	−0.065	−0.064
	*P*-value	0.049	0.049	—	—	0.039	0.039	0.039

**Table 2D:** Parameters of models assessing the impact of sleep duration on properties of individual networks in nonwhite and nonobese subcohort (*n* = 1406). Regression coefficients have been standardized to allow for comparisons between measures and *P*-values have been adjusted for false discovery

Network	Parameter	Efficiency	Global clustering	Median conn. (in)	Median conn. (out)	Modularity	Topological robustness	Topological stability
Left hemisphere								
Central visual	Beta	−0.079	−0.074	−0.081	−0.060	0.080	−0.081	−0.083
	*P*-value	0.010	0.016	0.010	0.050	0.010	0.010	0.010
Peripheral visual	Beta	−0.086	−0.085	—	—	0.101	−0.090	−0.086
	*P*-value	0.007	0.007	—	—	0.006	0.007	0.007
Dorsal attention	Beta	−0.104	−0.100	-	−0.065	0.095	−0.098	−0.096
	*P*-value	0.002	0.002	—	0.038	0.002	0.002	0.002
Limbic	Beta	−0.074	−0.064	—	—	—	−0.073	−0.065
	*P*-value	0.046	0.046	—	—	—	0.046	0.046
Fronto-parietal control	Beta	−0.078	−0.087	−0.068	—	0.063	−0.079	−0.078
	*P*-value	0.012	0.012	0.029	—	0.029	0.012	0.012
Default	Beta	−0.082	−0.085	−0.083	—	0.069	−0.089	−0.089
	*P*-value	0.008	0.007	0.009	—	0.022	0.007	0.007
Right hemisphere								
Central visual	Beta	−0.078	−0.077	−0.069	−0.063	0.094	−0.090	−0.090
	*P*-value	0.013	0.013	0.028	0.041	0.008	0.008	0.008
Peripheral visual	Beta	−0.065	−0.071	—	—	0.073	−0.075	−0.073
	*P*-value	0.047	0.034	—	—	0.034	0.034	0.034
Dorsal attention	Beta	−0.104	−0.109	−0.073	—	0.107	−0.107	−0.106
	*P*-value	0.001	0.001	0.018	—	0.001	0.001	0.001
Fronto-parietal control	Beta	−0.087	−0.085	−0.075	—	0.068	−0.087	−0.086
	*P*-value	0.006	0.006	0.019	—	0.021	0.006	0.006
Default mode	Beta	−0.069	−0.073	−0.066	—	0.070	−0.079	−0.078
	*P*-value	0.028	0.028	0.039	—	0.028	0.026	0.026

**Table 3:** Parameters of models assessing the impact of difficulty getting to sleep at night on properties of individual networks, for the entire cohort (A) and separate for nonobese participants (B). Regression coefficients have been standardized to allow for comparisons between measures and *P*-values have been adjusted for false discovery. Median connectivity is reported within each network (in) as well as between nodes belonging to the network and the rest of the brain (out)

Network	Parameter	Efficiency	Global clustering	Median conn. (in)	Median conn. (out)	Modularity	Topological robustness	Topological stability
A. Entire cohort (*N* = 5566)								
Left hemisphere								
Somatomotor	Beta	−0.041	−0.042	−0.035	−0.035	0.037	−0.044	−0.044
	*P*-value	0.004	0.004	0.013	0.014	0.009	0.004	0.004
Right hemisphere								
Somatomotor	Beta	−0.039	−0.039	−0.041	−0.040	0.038	−0.044	−0.046
	*P*-value	0.005	0.005	0.005	0.005	0.005	0.002	0.002
Limbic	Beta	−0.028	−0.026	−0.030	−0.033	0.046	−0.031	−0.033
	*P*-value	0.040	0.049	0.040	0.040	0.009	0.040	0.040
**B. Nonobese participants** **(*N* = 4802)**								
Right hemisphere								
Somatomotor	Beta	−0.037	−0.038	−0.033	−0.034	0.036	−0.041	−0.042
	*P*-value	0.015	0.014	0.028	0.028	0.017	0.014	0.01
Left hemisphere								
Somatomotor	Beta	−0.037	−0.038	−0.042	−0.039	0.039	−0.042	−0.044
	P-value	0.011	0.011	0.008	0.011	0.011	0.008	0.008

The same properties were negatively affected by snoring in temporo-parietal and limbic networks (*P* < 0.04) bilaterally. These results are summarized in Table 4 for the entire cohort (A) and nonwhite participants (B). Nonsignificant associations were estimated in the nonobese subcohort, suggesting that the effects may be the result of complex coupling between unhealthy BMI and the connectome as well as snoring and BMI (Table 1). In nonwhite participants, increased frequency of snoring was associated with decreased efficiency, global clustering, robustness, and topological stability in bilateral ventral attention/salience, limbic, frontro-parietal control, and temporo-parietal networks bilaterally, the left DM (except efficiency) and the right dorsal attention network (*P* ≤ 0.05). Following the adjustment for asthma, additional previously significant negative associations between frequency of snoring and global efficiency, clustering, robustness, and topological stability in the left DM, bilateral fronto-parietal control and bilateral ventral attention networks were no longer significant (*P* > 0.10).

**Table 4:** Parameters of models assessing the impact of snoring on properties of individual networks, for (A) the entire cohort (*n* = 5566) and (B) nonwhite participants (*n* = 1756). Regression coefficients reported here have been standardized to allow for comparisons between measures and *P*-values have been adjusted for false discovery. Median connectivity is reported within each network (in) as well as between nodes belonging to the network and the rest of the brain (out)

Network	Parameter	Efficiency	Global clustering	Median conn. (in)	Median conn. (out)	Modularity	Topological robustness	Topological stability
A. Entire cohort (*N* = 5566)								
Left hemisphere								
Temporo-parietal	Beta	−0.034	−0.036	—	—	0.049	−0.036	−0.036
	*P*-value	0.028	0.028	—	—	0.006	0.028	0.028
Limbic	Beta	−0.043	−0.044	—	—	0.044	−0.045	−0.048
	*P*-value	0.004	0.003	—	—	0.004	0.003	0.003
Right hemisphere								
Temporo-parietal	Beta	−0.038	−0.040	—	—	0.039	−0.034	−0.034
	*P*-value	0.025	0.025	—	—	0.025	0.033	0.033
Limbic	Beta	−0.049	−0.046	—	—	—	−0.046	−0.045
	*P*-value	0.002	0.002	—	—	—	0.002	0.002
**B. Non-white participants (*N* = 1756)**								
Left hemisphere								
Ventral attention/salience	Beta	−0.063	−0.070				−0.058	−0.056
	*P*-value	0.042	0.038				0.042	0.042
Limbic	Beta	−0.073	−0.069				−0.058	−0.058
	*P*-value	0.020	0.020				0.045	0.045
Fronto-parietal control	Beta	−0.071	−0.070	−0.056		0.066	−0.076	−0.073
	*P*-value	0.006	0.007	0.037		0.009	0.006	0.006
Default mode	Beta		−0.059				−0.056	−0.057
	*P*-value		0.050				0.050	0.050
Temporo-Parietal	Beta	−0.066	−0.067	−0.053	−0.058		−0.072	−0.071
	*P*-value	0.019	0.019	0.040	0.035		0.017	0.017
Right hemisphere								
Dorsal attention	Beta	−0.072	−0.073				−0.065	−0.063
	*P*-value	0.017	0.017				0.021	0.022
Ventral attention/salience	Beta	−0.064	−0.071	−0.061			−0.068	−0.066
	*P*-value	0.017	0.017	0.025			0.017	0.017
Limbic	Beta	−0.075	−0.066				−0.065	−0.061
	*P*-value	0.023	0.028				0.028	0.028
Fronto-parietal control	Beta	−0.063					−0.062	−0.062
	*P*-value	0.029					0.029	0.029
Temporo-parietal	Beta	−0.083	−0.079	−0.079	−0.055	0.082	−0.090	−0.091
	*P*-value	0.002	0.001	0.003	0.039	0.002	0.001	0.001

Difficulty breathing was also negatively associated with these properties in temporo-parietal and somatomotor networks bilaterally in the entire cohort (*P* < 0.03), the somatomotor network in nonwhite (but not in white) participants (*P* ≤ 0.01) and similarly in nonobese (*P* < 0.04), and the right temporo-parietal network in this subcohort (*P* ≤ 0.03). Similarly to snoring, following the adjustment for asthma, additional previously significant associations between difficulty breathing and left DM and left dorsal attention network properties were no longer significant (*P* > 0.05).

Finally, frequent difficulty waking up was associated with decreased efficiency, global clustering, robustness, and stability in bilateral dorsal attention networks (*P* ≤ 0.02) in nonwhite (but not in white) participants. These effects, and additional adverse modulations of networks properties of the right central visual, limbic, fronto-parietal control, and DMN (p ≤ 0.04) were also estimated in nonwhite and nonobese participants, suggesting that they may be BMI-independent. The dorsal attention network was similarly affected by the frequency of waking up tired and daytime sleepiness in nonwhite (*P* ≤ 0.04) as well as no-white and nonobese but not in white participants.

**Figure 1:**
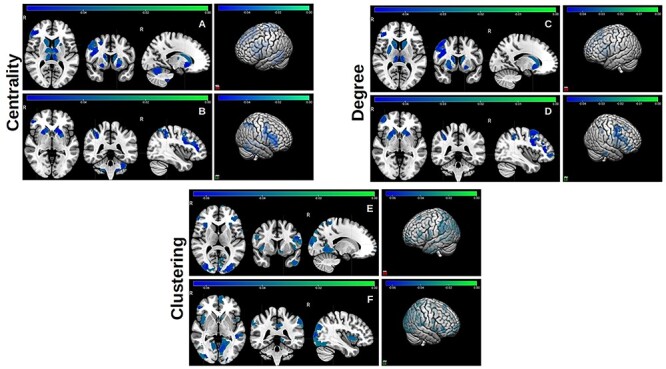
Significant negative effects of short sleep duration on node centrality, degree, and clustering. The colorbar represents the range of standardized regression coefficient values in statistical models that assessed these effects. Two and three-dimensional views (right panels) of both hemispheres are shown. Two sets of coronal, horizontal, and sagittal slices (left and right hemispheres) are included in top and bottom panels.

**Figure 2:**
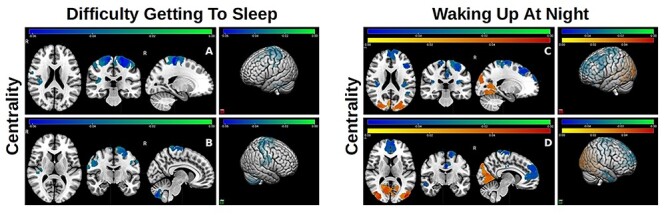
Significant negative (green/blue) and positive (orange/red) effects of difficulty getting to sleep and waking up more than twice per night on node centrality. The colorbar represents the range of standardized regression coefficient values in statistical models that assessed these effects. Two- and three-dimensional views (right panels) of both hemispheres are shown. Two sets of coronal, horizontal, and sagittal slices (left and right hemispheres) are included in top and bottom panels (left).

### Effects of Sleep Duration and Quality on Properties of Individual Brain Regions (Network Nodes)

The effects of sleep on local network properties, that is, those of individual nodes (the most granular scale), were finally examined, focusing on node degree (number of connections), centrality (topological role in the network), and local clustering.

**Figure 3:**
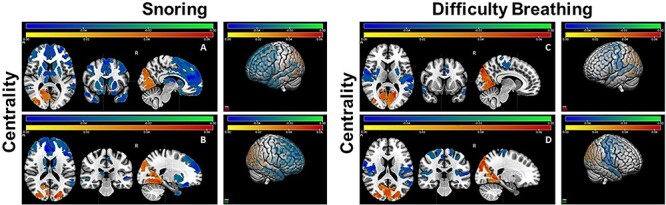
Significant negative (green/blue) and positive (orange/red) effects of snoring and difficulty breathing on node centrality. The colorbars represent the range of standardized regression coefficient values in statistical models that assessed these effects. Two- and three-dimensional (right panels) views of both hemispheres are shown. Two sets of coronal, horizontal, and sagittal slices (left and right hemispheres) are included in top and bottom panels (left).

I) Sleep duration: Shorter sleep duration was negatively associated with node properties in distributed brain regions (Fig. 1). These included distributed posterior areas that are part of the ventral attention/salience and dorsal attention networks, elements of the limbic network (adversely affected local clustering), and thalamic structures (adversely affected node centrality and degree). Together these areas and structures are part of a distributed network that includes the insula (adversely affected node clustering) and frontal cortical regions (adversely affected node degree partially in the dorsolateral prefrontal cortex) and support emotional processing and regulation. Shorter sleep duration was also associated with lower node centrality in the cerebellum and nodes that are part of the DMN. These effects were also examined separately in nonobese participants. The same results were obtained for node centrality and clustering across the same brain regions, but the effects on node degree were no longer significant, which suggests that the number of connections in these brains areas may be adversely modulated by complex coupling between sleep duration and unhealthy BMI. To examine the joint effects of BMI and insufficient sleep, models were also developed with the interaction between the two parameters as the primary predictor. The same negative effects were estimated across brain regions and node properties, with extensive additional effects on distributed bilateral anterior regions, overlapping with elements of the DMN and fronto-parietal control networks, which has been shown to be affected by BMI independently of sleep as well (Brooks et al. 2021).
II) Sleep quality also had significant effects on local network properties, which are summarized in Figures 2 and 3. Frequent difficulty getting to sleep had a significant negative effect on node centrality primarily in the ventral attention/salience network. Waking up more than twice per night had similar adverse effects on these and also parts of the fronto-parietal control network. In addition, frequency of these events with positively associated with posterior visual network regions (Fig. 2). When the nonobese subcohort was examined, approximately the same negative and aberrant positive effects of waking up more than twice per night on node centrality were estimated, but the effects of difficulty getting to sleep were no longer significant.

Snoring had extensive negative effects on node centrality in bilateral frontal and anterior temporal areas and difficulty breathing had similar impacts in a subset of the same regions (Fig. 3). Both snoring and difficulty breathing were associated with increased node centrality in the same posterior regions of visual networks as waking up at night, suggesting alteration of the importance of nodes in these areas as a result of disrupted and/or fragmented sleep. When these effects were examined in the nonobese subcohort, snoring and difficulty breathing were associated with increased centrality in the same posterior regions identified in the entire cohort, but the extent of negative snoring effects on frontal regions were substantially reduced and were more localized to anterior parts of the frontal lobe, which suggests dependence of some but independence of other effects from unhealthyBMI.

## Discussion

This first-of-its-kind study, in sample size, comprehensive evaluation of multiple aspects of sleep and multiscale assessments of the topology of developing functional brain networks, has investigated the impact of sleep on the organization of the connectome in early adolescence, including networks that support higher level processes that continue to evolve during this complex developmental period. Shorter duration and reduced quality sleep are both major contributors to unhealthy sleep, a growing public health problem in modern societies. In children, unhealthy sleep may have profound short- and long-term adverse effects. Specifically, it may be detrimental to the developmental of critical brain circuits that support decision-making, problem solving, executive control, and social cognition. These circuits undergo significant maturation and reorganization in adolescence and are vulnerable to repeated sleep restriction and frequent episodes of disordered/disrupted sleep. Understanding the effects of shorter duration and reduced quality sleep on functional circuits in the developing brain is paramount to elucidating the neural basis and developmental trajectories of widely reported decreased academic performance, deficits in attention and sensory processing, and impaired learning in children who do not obtain sufficient high-quality sleep (Curcio et al. 2006).

Using resting-state fMRI data from 5566 early adolescents from the ABCD cohort and corresponding data on sleep habits from the SDSC, this study has estimated the effects of sleep duration and multiple aspects of sleep quality on fundamental topological characteristics of functional networks across spatial scales, from individual regions (nodes) to large-scale circuits and the entire brain. Prior investigations of structural MRI from this and other smaller pediatric cohorts have identified significant gray matter changes as a result of insufficient sleep and/or sleep-disordered breathing (Philby et al. 2017; Sung et al. 2020; Isaiah et al. 2021). However, relatively few studies, primarily in smaller cohorts and focusing mostly on measures of connectivity, have examined modulations of functional networks by sleep across spatial scales of topological organization. Connectivity only reflects the strength of connections between brain regions but does not measure their organizational principles, which are critical to cognitive processing. To address this important gap in knowledge, this study has specifically studied the association between sleep and topological properties on which the efficiency of neural information processing, the brain’s flexibility, adaptability and resilience, and ultimately cognitive performance critically dependon.

Sleep duration and quality were significantly associated with demographics, BMI, and screen time. Girls slept significantly less, took a longer time to fall asleep, and were more frequency anxious or afraid when falling asleep than boys. In addition, they had difficulty waking up in the morning, were tired when walking up and felt sleepy during the day more often than boys. They also had significantly higher BMI (but no significant differences in age or pubertal stage), which was associated with shorter sleep duration, more frequent movement during the night, sweating, snoring, difficulty waking up, and daytime sleepiness. On the other hand, boys sweated excessively during the night more often than girls. These findings may reflect previously reported significant sex differences in adolescent sleep physiology (Markovic et al. 2020). In addition, nonwhite youth slept significantly less (on average 8–9 h) and snored more frequently than white youth (who slept on average 9–11 h), although the latter had more difficulty getting to sleep and were more often anxious when falling asleep. In addition, Hispanic youth slept significantly less than non-Hispanic youth (8–9 h vs. 9–11 h) but were less reluctant to go to bed, had less difficulty getting to sleep at night, and felt tired when waking up and sleepy during the day less often than nonHispanic youth. These results are in agreement with prior findings from a number of studies that have reported sleep disparities (particularly in sleep duration) in children from racial and ethnic minority groups (Hawkins and Takeuchi 2017; Guglielmo et al. 2019).

Sleep duration and quality, including reluctance to go to bed, difficulty getting to sleep, increased movement during sleep and dream-like scenes while falling asleep, difficulty falling asleep after awakening during the night, more frequent snoring, excessive sweating, difficulty waking up, and daytime sleepiness, were negatively associated with screen time. These results confirm those from earlier studies, including recent ones based on the ABCD cohort and thus children in early adolescence, showing a direct association between screen media use and sleep disturbance severity (Hisler et al. 2020; Kirlic et al. 2021). They also highlight the rapidly growing problem of youth spending substantial time on screen media, which has detrimental implications for fundamental aspects of health such as BMI (Brooks et al. 2021), sleep duration, and quality.

The impact of sleep duration and quality on the topology of the functional connectome was investigated at three spatial scales: the entire brain, multiple resting-state networks, and individual regions (network nodes). Similar results were estimated across these scales, reflecting widespread negative associations between connectome properties and shorter sleep duration and/or lower sleep quality, from localized brain regions (network nodes) performing domain-specific computations to large-scale networks that integrate information across brain regions. Specifically, reduced sleep duration was consistently associated with lower efficiency, lower global topological clustering, reduced robustness/resilience to external perturbations, and lower topological stability of the entire brain (as a single network), as well as bilateral dorsal and ventral attention, salience and fronto-parietal networks, and the DMN. Reduced connectivity, centrality (topological importance of a region/node), clustering and/or number of node connections in these networks as well as the thalamus, basal ganglia, parts of the hippocampus (dentate gyrus) and parts of the cerebellum, were also estimated. These results suggest that shorter sleep duration may affect both local and long-range connections, and consequently both segregated processing and transmission of information and integration across the brain.

These findings have important implications for cognitive function in the early adolescent brain. The fronto-parietal network (including parts of the dorsolateral and ventrolateral prefrontal cortex and parts of the posterior parietal cortex) plays a critical role in executive control, integration of information for action planning, working memory, and other fundamental processes such as pain processing (Kong et al. 2013; Zanto and Gazzaley 2013; Marek 2018). Based on their extensive connections and role in cognitive function, brain regions that are part of this network are considered connectome hubs, that is, critical regions that are involved in multiple tasks and integrate information across brain areas, inputs and processes. Reduced efficiency and robustness of this network may have profound effects on higher-level cognitive processes that are still evolving in adolescence, and may lead to impaired decision-making and increased risk behaviors. Similarly, deficits in attention, as a result of impaired organization of the neural circuitry that supports it, may have a significant impact on sensorimotor function, motor planning, and sensory processing (Vossel et al. 2014). In children, it may also have broad implications for learning and academic performance (Curcio et al. 2006).

The efficiency, community organization (reflected in global clustering), robustness, and topological stability of the DMN were also negatively associated with sleep duration. The DMN is strongly coordinated at rest, but its activity decreases substantially during task performance. Recent research has also shown that the (temporal) stability of the DMN predicts task engagement (McCormick and Tezler 2018). Given the ubiquitous role of this network in cognition function and mental health (Mars et al. 2012; Whitfield-Gabrieli and Ford 2012; Raichle 2015), and its incomplete maturation in adolescence (Fair et al. 2008), shorter sleep duration may have profound and potentially long-lasting negative effects on cognitive and mental health. In addition, reduced local network properties in the thalamus, which is central to the function of the DMN, were also associated with shorter sleep duration. Similar negative associations were identified in the basal ganglia, parts of the hippocampus and the cerebellum, which, together with adverse effects in cortical network properties may result in widespread deficits in executive control, sensorimotor function, memory processes, and emotion regulation (Haber and Calzavara 2009; Pessoa 2017). Of note is that many of these topological modulations were estimated in nonobese participants as well, which suggests that they may be directly associated with shorter sleep duration, instead of an indirect effect of unhealthy BMI. Finally, widespread negative network effects were estimated in nonwhite (as well as nonwhite and nonobese) but not in white participants, highlighting disproportionately disparate effects of shorter sleep duration on the connectome in racial minority groups.

Beyond sleep duration, multiple aspects of sleep quality were significantly associated with local and network-wide connectome properties, partially in overlapping networks and structures as those impacted by shorter sleep duration. The somatomotor, limbic, the DMN, fronto-parietal control, and attention networks were the ones mostly impacted by the frequency of reduced quality sleep. Increased sleep latency was associated with reduced efficiency, global clustering, node centrality, robustness, and topological stability of the somatomotor network bilaterally (both the entire cohort and nonobese subcohort), dorsal attention, and limbic networks. Some of these effects were no longer significant in the nonobese subcohort, suggesting potential coupling between reduced sleep quality and BMI. Waking up more than twice per night was associated with reduced topological importance (centrality in the network) of bilateral areas of the DMN, left fronto-parietal and bilateral somatomotor networks but was associated with abnormally increased node centrality in posterior bilateral visual areas. Given that elements of the DMN are considered network hubs, such topological modulations may have extensive negative implications for cognitive function across domains. Aberrantly increased local connectivity and number of node connections (and thus its topological importance in the network) at rest may adversely affect the brain’s flexibility to respond to sensory inputs (e.g., visual) and coordinate the functional activity of distributed networks in response cognitive demands. Reduced centrality of DMN hubs may lead to impaired integration of information from distributed brain regions, a key aspect of consciousness, perception, and cognitive function across domains (Tononi 2004; Power et al. 2011; Oldham and Fornito 2019).

Additional negative associations between reduced sleep quality and network properties were estimated specifically in nonwhite participants. Increased frequency of difficulty waking up in the morning, waking up tired and/or daytime sleepiness was associated with reduced network efficiency, global clustering, robustness, and topological stability of the dorsal attention network in nonwhite (but not in white) participants. Thus, beyond shorter sleep duration, lower quality sleep may also have disparate detrimental effects on connectome properties in racial minority groups.

Sleep-disordered breathing (frequent difficulty breathing during sleep and snoring) was negatively associated with multiple properties across networks. Frequent snoring was negatively associated with the efficiency, clustering, topological robustness, and stability of temporo-parietal and limbic networks. In nonwhite participants, additional negative associations were estimated in bilateral ventral attention, fronto-parietal control, temporo-parietal, left DM, and right dorsal attention. Increased frequency of sleep-disordered breathing was also associated with reduced node centrality in the thalamus and parts of the basal ganglia, but increased centrality in posterior visual areas. Prior work has reported aberrant (including increased) connectivity in these areas in individuals with OSA (Schultz et al. 2000; Khazaie et al. 2017) and sleep disorders such as narcolepsy (Fulong et al. 2020). Both the thalamus and basal ganglia play central roles in cognitive processes, including those still developing in adolescence, for example, executive function and decision-making, but are also part of the distributed reward system. The basal ganglia are critical to this system’s normal function and thalamic structures for its regulation (Koob and Le Moal 2005; Halassa and Kastner 2017). Aberrant changes in the properties of this network, particularly the topological importance of hubs such as the basal ganglia and the thalamus may result in substantial deficits in neural information processing and integration, but may also lead to mental health issues across domains and risk behaviors, including substance use (Naranjo et al. 2001; Schneider et al. 2012).

Despite its strengths, including the analysis of multiscale network properties, large sample size and comprehensive investigation of different aspects of sleep, this study also has some limitations. First, children with a history of bipolar disorder and diagnosed ADHD were excluded from the cohort and analyses were adjusted for overall history of anxiety and depression. A recent genomic study using data from the ABCD study has shown that increased genetic susceptibility to these disorders may adversely impact sleep health (Ohi et al. 2021). Although neither history of depression nor anxiety had a significant effect on any of the results, an extensive investigation of other mental health issues and their impact on sleep was not conducted, as it was beyond the scope of this study. Therefore, it is possible that some findings could be confounded by such underlying disorders, although there is increasing evidence of a bidirectional relationship between sleep and mental health (Alvaro et al. 2013). All analyses were adjusted for asthma (which was significant in a number of models, including questions related to sleep disordered breathing), but there was no information on diagnosis of sleep disorders for any participant. It is possible that some children in this cohort may have been diagnosed with such a disorder, although the number is likely to be low and unlikely the significantly affect the findings in this study.

In addition, this study did not examine actigraphy data, which at the time of analysis were available from a subset of ~50% of the entire ABCD cohort. Instead, it focused on the SDSC, which was available for all participants. Given that this study excluded participants based on neuroimaging data quality, clinical MRI findings and history of bipolar disorder or ADHD, actigraphy data would have probably been available only for a small subset of included participants, which would limit further stratification of the analyses, for example, to decouple the effects of race and/or BMI. Although the SDSC is an indirect and subjective tool for measuring sleep, it provides a comprehensive assessment of sleep quantity and quality, is widely used and is particularly suitable for collecting sleep-related information in large cohorts. All its items were investigated in the study, although only few were found to be significantly associated with network properties. Another limitation is that the SDSC asked participants to report sleep habits in the past 6 months but not the night prior to their fMRI scan. Furthermore, the study could not examine other sleep measures such as social jetlag, that is, the difference in sleep time between school and weekend/free days. These data were collected in the ABCD study, using the Munich Chronotype questionnaire, but at the 2-year follow up not at baseline (the assessment point analyzed here).

Another limitation is that identified resting-state (and thus task-independent) network effects could not be directly mapped onto behavioral/cognitive performance deficits. However, given that resting-state networks such as the DMN, limbic and fronto-parietal control networks are ubiquitously involved in cognitive function and processes that are still developing in adolescence, the reported aberrant topological modulations by sleep may lead to widespread cognitive deficits and mental health issues. In addition, sleep-disordered breathing is common early in life (age ≤ 6 years), and prior work has shown that snoring in early childhood may predict cognitive performance in adolescence (Gozal and Pope 2001). Thus, damage to the functional brain circuitry may have occurred long before children were measured in the ABCD study, but history of sleep disordered breathing and potential early interventions (e.g., adenotonsillectomy) was not available. Finally, both connectome organization and sleep patterns may be independently affected by other experiential, environmental and genetic factors. These may mediate or moderate the relationship between sleep and network properties, in addition to their direct effects on each other. Given multimodal data, including genetic and environmental, in the ABCD study, a future investigation can systematically assess these complex indirect relationships.

Despite some limitations, this study makes a significant contribution to our currently incomplete understanding of the complex relationship between sleep and developing brain, particularly sleep-related modulations of the topological organization of incompletely maturated and evolving functional circuitry, on which information processing and cognitive performance ubiquitously depend. Findings suggest that shorter duration and lower quality sleep are negatively associated with topological network properties across spatial scales of organization. Together, these networks play a fundamental role in cognition, including in higher level processes such as executive function, but are vulnerable to stressors in adolescence. Thus, aberrant modulations of their organization may lead to cognitive deficits, increased risk behaviors, and mental health issues. This study also highlights that shorter duration and reduced quality sleep may have disproportionately disparate connectome effects in children from racial minority groups.

## Supplementary Material

SupplementalMaterials_fMRIAnalysis_CerebralCortex_tgab062

## Notes

The authors would like to thank Sean Parks for his work on the fMRI analysis pipeline, and Dr Raffaele Potami and the research computing team at Harvard Medical School for their help in the development of the NGNDA pipeline. *Conflict of Interest*: None declared.
