# Supplementary material for: Shorter Duration and Lower Quality Sleep Have Widespread Detrimental Effects on Developing Functional Brain Networks in Early Adolescence
Source: Cereb Cortex Commun. 2021 Oct 26;3(1):tgab062. doi: 10.1093/texcom/tgab062 (PMC8759437; doi:10.1093/texcom/tgab062)
Supplement: SupplementalMaterials_fMRIAnalysis_CerebralCortex_tgab062 [file supplementalmaterials_fmrianalysis_cerebralcortex_tgab062.doc]

**S. Supplementary Materials**

**S1. fMRI preprocessing using the NGNDA pipeline** The fMRI part of the Next-Generation Neural Data Analysis (NGNDA) pipeline combines the functionality of the SPM12 software package (Friston et al, 1995, 2007) with custom Matlab codes. It provides the flexibility to make data-specific decisions on denoising, includes signal decompositions and implements multiple parcellation and connectivity estimation approaches.

The SPM12 component of the pipeline includes structural segmentation, initial fMRI frame removal, coregistration of fMRI to structural MRI, slice-time correction, and normalization to MNI152 space. Each participant’s resting-state fMRI segment (run) was processed as follows: Rigid body motion parameters, estimated from provided realignment measures (Power et al., 2012), were bandstop-filtered to suppress breathing artifacts in the range 0.28-0.46 Hz; Fair et al, 2020). Frames with displacement (an estimate of overall head motion) >0.3 mm are censored (Power et al, 2012; Siegel et al, 2014). Runs with <10.0% of frames censored due to excessive motion are further analyzed. Once motion was filtered out of each (zero-mean) voxel time series using the statistical modeling approach in (Power et al, 2014), residual signals are bandpass-filtered in the frequency range of physiologically relevant BOLD signal content (0.01-0.25 Hz; Yuen et al, 2019). Time points with amplitudes exceeding the upper extreme outlier amplitude statistic of each voxel time series are assumed to be artifacts and are set to the median amplitude of the voxel time-series while retaining their original sign.

Multiple parcellation options are available in the pipeline. In the present study, parcellation based on the Schaefer-1000 cortical atlas (Yeo et al, 2011; Schaefer et al, 2018), Melbourne subcortical atlas (Tian et al, 2020), and probabilistic MR atlas of the human cerebellum (Diedrichsen et al, 2009) was used to map the voxels in normalized MNI space onto 1088 brain regions (1000 cortical, 54 subcortical and 34 cerebellar). Within each region, the time series of all voxels were averaged. Given that neuroimaging data in the ABCD study were collected with different scannes across sites and consequently there were significant scanner effects on signal amplitudes, all data are normalized. For each time series their respective median absolute amplitude was first estimated. The overall median (over the number of parcels) was used to normalize each time series so that they are comparable between brains. The unsupervised Ensemble Empirical Mode Decomposition (EEMD; Torres et al, 2011) was then used to estimate a small set of narrowband components (modes) from each regional signal. Based on their characteristic frequencies and amplitudes, outlying components were eliminated and the remaining ones were linearly superimposed to obtain further denoised signals.

**S2. Connectivity matrix thresholding to estimate the adjacency matrix** Multiple cohort-level and brain-specific statistics were estimated from the correlation matrix, including the median, inter-quartile range (IQR), moderate and extreme outliers (median + 1.5*IQR; median + 3*IQR; Tukey, 1977) and their respective confidence intervals (CI). Assuming that functional connectivity at rest is relatively low (with the exception of a few networks such as the default-mode network, which are actively coordinated at rest), and to minimize the likelihood of spuriously increased connectivity associated with residual motion artifacts, the rs-fMRI with the lowest median connectivity was selected for further analysis. This often also coincided with the run with the lowest number of frames censored for motion. The upper CI of the moderate cohort-based outlier was applied as a common threshold to all connectivity matrices, to obtain corresponding weighted adjacency matrices. Thus, all connections below this threshold were set to 0. Although there are different ways of thresholding connectivity, each makes a number of assumptions and has advantages and shortcomings (Bordier et al, 2017; Del Ferraro, 2018). The choice of threshold may also reflect the biological question of interest e.g., a very conservative threshold such as the extreme outlier may lead to a sparse weighted adjacency matrix with only highly connected regions being non-zero. In this study several thresholds were compared, based on the median, 75^th^ percentile, moderate and extreme outlying connectivity values, as well as percolation-based threshold, (Esfahlani and Sayama, 2018), which was found to be approximately equal to the 75^th^ percentile of peak cross-correlation. The cohort-based moderate outlier was selected as a conservative connectivity threshold.

**S3. Description of estimated network measures**  *Mean* and *median connectivity* were estimated from the weighted adjacency matrix. For individual networks, mean and median connectivity were estimated within each network as well as between nodes belonging to the network and those outside the network. The *local (node-specific) clustering coefficient* was calculated as the ratio of a node’s neighbors that were neighbors themselves (Rubinov and Sporns, 2010) and *global clustering* was estimated as the mean of these local coefficients. *Modularity* measured the degree to which the parts of the network with connections above the threshold could be divided into non-overlapping node communities. Although it was primarily estimated using the Newman method (Newman, 2006), a second set was obtained using the Louvain method (Blondel et al, 2008). *Degree* was the number of non-zero connections for each node. *Global efficiency* was calculated as the average inverse shortest path length of the network and quantifies the efficiency of distant information transfer (Stanley et al, 2015). *Eigenvector centrality* measured node importance in the network. *Small-worldness* was calculated as the ratio of normalized global clustering to normalized characteristic path length, using the approach in (Bassett and Bullmore 2017) with 20 random graphs, generated from binary adjacency matrices (Anderson and Cohen, 2013). The topological organization of the network as a combination of highly connected node clusters (modules) communicating via sparse long-range connections was measured by the network’s small worldness (Watts and Strogatz, 1998; Telesford et al, 2011). *Network robustness* was measured by *natural connectivity,* calculated as the average eigenvalue of the weighted adjacency matrix (Wu et al, 2011). Finally, *topological network stability* was estimated using the largest eigenvalue of the adjacency matrix (Restrepo et al, 2007).

**References**

Anderson A, Cohen MS (2013). Decreased small-world functional network connectivity and clustering across resting state networks in schizophrenia: an fMRI classification tutorial. *Front Human Neurosci*, *7*: 520.

Blondel, VD, Guillaume, J, Lambiotte, R, Lefevre, E. Fast unfolding of communities in large networks. *J Stat Mech*. 2008. P10008

Bordier C, Nicolini C, Bifone, A (2017), Graph Analysis and Modularity of Brain Functional Connectivity Networks: Searching for the Optimal Threshold, Front Neurosci, 11: 441.

Del Ferraro G., Moreno A., Min, B. et al (2018). Finding influential nodes for integration in brain networks using optimal percolation theory. Nat Commun 9, 2274.

Diedrichsen J, Balsters JH, Flavell J, et al (2009). A probabilistic MR atlas of the human cerebellum, NeuroImage, 46 (1): 39-46.

Drollette ES, Scudder MR, Raine LB, et al (2014), Acute exercise facilitates brain function and cognition in children who need it most: An ERP study of individual differences in inhibitory control capacity. Dev Cog Neurosci, 7: 53-64.

Esfahlani FZ, Sayama H (2018), A Percolation-Based Thresholding Method with Applications in Functional Connectivity Analysis, International Workshop on Complex Networks, CompleNet 2018: Complex Networks IX, 221-231.

Fair DA, Miranda-Dominguez O, Snyder AZ, et al (2020). Correction of respiratory artifacts in MRI head motion estimates. NeuroImage, 208: 116400.

Friston KJ, Holmes AP, Worsley KJ et al (1995), Statistical Parametric Maps in functional imaging: A general linear approach, Human Brain Mapp, 2:189-210.

Friston KJ, Ashburner JT, Kiebel SJ, et al (2007). Statistical Parametric Mapping: The Analysis of Functional Brain Images, Academic Press.

Newman, MEJ. Modularity and community structure in networks. *Proc Natl Acad Sci*. 2006. 103(23):8577-8582.

Power JD, Barnes KA, Snyder AZ, et al (2012). Spurious but systematic correlations in functional connectivity MRI networks arise from subject motion. NeuroImage,​ 59 (3): 2142-2154.

Power JD, Mitra A, Laumann TO, et al (2014). Methods to detect, characterize, and remove motion artifacts in resting state fMRI. NeuroImage, 84: 320–341.

Restrepo, JG, Ott, E, Hunt, BR (2007), Approximating the largest eigenvalue of network adjacency matrices, Phys Rev E, 76: 056119.

Rubinov M, Sporns O. (2010). Complex network measures of brain connectivity: Uses and interpretations. NeuroImage 52: 1059-69.

Schaefer A, Kon, R, Gordon EM, et al (2018), Local-Global Parcellation of the Human Cerebral Cortex from Intrinsic Functional Connectivity MRI.​ Cereb Cortex​,28 (9): 3095-3114.

Siegel JS, Power JD, Dubis JW, et al (2014), Statistical improvements in functional magnetic resonance imaging analyses produced by censoring high-motion data points. ​Human Brain Mapping, ​35 (5): 1981-1996.

Stanley ML, Simpson SL, Dagenbach D, et al (2015). Changes in brain network efficiency and working memory performance in aging. *PLoS One*, 10(4): e0123950.

Telesford QK, Joyce KE, Hayasaka S, et al (2011). The ubiquity of small-world networks. *Brain Connect*, 1(5): 367-375.

Tian Y, Margulies DS, Breakspear M, et al (2020).Topographic organization of the human subcortex unveiled with functional connectivity gradients. ​Nat Neurosc, 23 (11): 1421-1432.

Torres ME, Colominas MA, Schlotthauer G, et al (2011). A Complete Ensemble Empirical ModeDecomposition with Adaptive Noise. ​IEEE Int Conf Acoust on Speech and Signal Proc.​, ICASSP-11: 4144-4147.

Trudeau F, Shephard RJ. (2009). Relationships of Physical Activity to Brain Health and the Academic Performance of Schoolchildren. American Journal of Lifestyle Medicine, 4(2), 138-150.

Tukey JW (1977), Exploratory data analysis. Addison-Wesely.

Van de Sande-Lee S, Pereira FRS, Cintra DE et al (2011), Partial Reversibility of Hypothalamic Dysfunction and Changes in Brain Activity After Body Mass Reduction in Obese Subjects.

Voss MW, Erickson KI, Prakash RS, et al (2013). Neurological markers of exercise-related brain plasticity in older adults. Brain Behav Immun, 28: 90-99.

Voss MW, Vivar C, Kramer AF, et al (2013). Bridging animal and human models of exercise-induced brain plasticity. Trends Cogn Sci, 17(10), 525-544.

Watts DJ, Strogatz SH (1998), Collective dynamics of “small-world” networks. Nature. 393 (6684):440–2.

Wu, J., M. Barahona, Y. Tan, H. Deng. (2011). Spectral Measure of Structural Robustness in Complex Networks. *IEEE Transactions on Systems, Man, and Cybernetics - Part A: Systems and Humans*, *41*(6), 1244–1252.

Yeo BTT, Krienen FM, Sepulcre J (2011), The organization of the human cerebral cortex estimated by intrinsic functional connectivity, J Neurophysiol, 106: 1125–1165.

Yuen NH, Osachoff N, Chen JJ (2019). Intrinsic frequencies of the resting-state fMRI signal: The frequency dependence of functional Connectivity and the effect of mode mixing. ​Frontiers Neurosci,​ 13: 900.
